# Association between Hepatic Steatosis and Entecavir Treatment Failure in Chinese Patients with Chronic Hepatitis B

**DOI:** 10.1371/journal.pone.0034198

**Published:** 2012-03-30

**Authors:** Xi Jin, Yi-peng Chen, Yi-da Yang, You-ming Li, Lin Zheng, Chuan-qing Xu

**Affiliations:** 1 Department of Gastroenterology, The First Affiliated Hospital, College of Medicine, Zhejiang University, Hangzhou, China; 2 Department of Infectious Disease, The First Affiliated Hospital, College of Medicine, Zhejiang University, Hangzhou, China; Duke University School of Medicine, United States of America

## Abstract

**Background:**

The coexistence of HBV infection and nonalcoholic fatty liver disease (NAFLD) becomes characteristic of liver disease in China, with unknown bilateral influence. We aimed to investigate the effect of hepatic steatosis, a common hepatocyte change in NAFLD, on antiviral therapy in patients with chronic hepatitis B (CHB).

**Methods and Findings:**

We carried out a prospective nested case control study in CHB patients receiving Entecavir for initial antiviral therapy, by recording demographic, anthropometric and clinical data at baseline, 24^wk^, 48^wk^ and 96^wk^. Univariate analysis and multivariate logistic regression were applied to find out independent factors of hepatic steatosis and Entecavir treatment failure. The rates of HBV-DNA clearance, HBeAg seroconversion and ALT normalization were compared between CHB patients with and without steatosis by post hoc analysis. A total of 267 Chinese patients with CHB entered final analysis, with overall percentages of hepatic steatosis and HBeAg positive as 30.5% and 62.4%. Multivariate analysis showed waist circumference, serum TG and uric acid levels were independent factors of hepatic steatosis. The response rates to Entecavir were 54.9%, 63.8%, 74.2% at 24^wk^, 48^wk^ and 96^wk^. Hepatic steatosis was revealed as an independent factor of Entecavir treatment failure by multivariate logistic regression at 24^wk^, 48^wk^ and 96^wk^. In CHB patients with hepatic steatosis, HBV-DNA clearance and HBeAg seroconversion were both lower throughout the follow-up, but only the former reached statistical significance. Besides, ALT normalization was also significantly lower at 24^wk^ and 48^wk^.

**Conclusion:**

Hepatic steatosis is significantly associated with Entecavir treatment failure and metabolic factors are independent factors of hepatic steatosis in CHB patients, which called for a specified antiviral strategy in CHB patients with NAFLD.

## Introduction

Hepatitis B virus (HBV) affects over 350 million people worldwide while countries in Asia and Africa account for over 70% of chronic HBV infection, with prevalence up to 15%–20% [1], [2]. In China, it was estimated that at least 10% of the general population are chronically infected with HBV, which becomes the most common cause of liver diseases [3]. Though the efficacy of antiviral therapy in chronic hepatitis B (CHB) has been greatly improved in the last decades after discovery of interferon and nucleoside analogues, lack of response still remains common [4]. It is well recognized that uncontrolled virus replication can cause liver damage and predispose those nonresponders into liver diseases of advanced stage. Therefore, unraveling factors associated with treatment failure in CHB patients is of clinical importance.

Nonalcoholic fatty liver disease (NAFLD) is defined as a common clinico- pathologic condition characterized by lipid deposition with/without inflammation in hepatocytes and comprises a wide spectrum of liver damage, including simple steatosis, nonalcoholic steatohepatitis (NASH) and fibrosis [5]. With social development and lifestyle change, NAFLD has now become a major cause of liver related morbidity and mortality, with the incidence of around 20% worldwide [6] and 15% in China [7]. Therefore, the coexistence of HBV infection and NAFLD becomes a novel characteristic of liver disease in China. However, their bilateral influence in both disease development and therapeutic response has been rarely reported.

Hepatic steatosis has long been considered as a common hepatocellular change in both simple steatosis and NASH. Recently, accumulated evidence showed that the frequency of hepatic steatosis in CHB patients ranged from 27% to 51%, higher than that in the general population, hinting its potential effects in CHB [8]. Moreover, steatosis in CHB patients seems to be a result of metabolic factors of the host rather than the effect of virus [9], [10]. Nevertheless, the effect of hepatic steatosis on treatment response in CHB patients is largely unknown. Therefore, we prospectively investigated, in an unmatched nested case control study of CHB patents receiving initial Entecavir therapy, the frequency of steatosis, its association with host and viral factors and its impact on the response to antiviral therapy.

## Methods

### Ethics statement

The protocol was approved by the institutional review board at Zhejiang University and conducted in accordance with the Declaration of Helsinki. The study design and manuscript preparation fully followed guideline from the STROBE statement [11]. All written informed consent was collected.

### Protocol

We have prospectively enrolled a cohort of CHB patients receiving Entecavir as initial antiviral therapy to investigate the drug's efficacy and side effects, from January 2007 till now in our hospital. Portion of the data between July 2007 and November 2009 were selected for analysis in this study. The dose of Entecavir was 0.5 mg/d per os with average follow-up of 79.3 weeks. Treatment was discontinued in the case of primary nonresponse and all side effects were registered. The enrollment criteria were mainly based on the Chinese official guideline for the treatment of CHB [12]: HBV-DNA≥10^5^ copies/mL in HBeAg (+) patients or HBV- DNA≥10^4^ copies/mL in HBeAg (−) patients; abnormal alanine aminotransferase (ALT) level ≥2 ULN (upper limit of normal range, 50 U/L); Age >18 y and never received anti-HBV therapy before this study. Exclusion criteria included: pregnant or on breast feeding; underwent hepatotoxic, steatogenic, antineoplastic, systemic immuno-modulator treatment within a period of 6 month before the start of antiviral therapy; coexistent with human immunodeficiency infection, autoimmune hepatitis, hepatocellular carcinoma, Wilson's disease, primary biliary cirrhosis, primary sclerosing cholangitis, HCV infection and other virus related hepatitis; neutrophil count <1500/mm^3^ or platelet count <100000/mm^3^; a history of psychiatric disease; and evidences of alcohol addiction from a well designed questionnaire [13] recording the frequency, type and amount of alcohol consumption (defined as alcohol intake ≥40 g/d in man and ≥20 g/d in women over 5 years; or alcohol intake >80 g/d within 2 weeks).

In this cohort, we compared the baseline demographic, anthropometric and serologic data between CHB patients with and without steatosis, aiming to find associated factors of hepatic steatosis. We then collected the patients' clinical and biochemical data at 24^wk^, 48^wk^ and 96^wk^. Thereafter, we divided patients into groups of response and nonresponse at different time spot and compared the patients' baseline characteristics. We used unmatched design in this nested case control study because all enrolled subjects had routine clinical and biochemical test so that we don't need matched controls to decrease research expenses. Finally, we retrospectively divided patients into groups with and without hepatic steatosis at baseline and then separately compared their HBV-DNA clearance, HBe seroconversion in HBeAg (+) patients and ALT normalization at above set time spots.

### Demographic, anthropometric and serologic data

On enrollment, a precompiled form was filled out to collect demographic and anthropometric data, including age, gender, body mass index (BMI, calculated as weight in kilograms divided by height in meters squared), waist circumference (measured midway between the lowest rib and the iliac crest), race, family history of HBV infection (defined as at least one of parents or siblings have HBV), hypertension (defined as a patient on antihypertensive drug for blood pressure over 140/90 mmHg) and diabetes mellitus (DM, defined as fasting glucose ≥7.0 mmol/L or with past history of diagnosed DM). Overweight and obese were defined as BMI≥25 kg/m^2^ and BMI≥30 kg/m^2^, according to the WHO definition. Hepatic steatosis was detected by ultrasound B examination.

At 24^wk^, 48^wk^ and 96^wk^, an overnight fasting blood sample was taken for routine analysis, including ALT, aspartate aminotransferase (AST), total bilirubin (TB), glutamyltranspeptidase (GGT), fasting blood glucose (FBG), alkaline phosphatase (ALP), cholesterol (Chol), triglyceride (TG), uric acid. HBsAg, HBeAg, anti-HBe, anti-HBc and HBsAb were detected by time resolved fluoroimmunoassay (TRFIA) on an Anytest TRFIA analyzer (SYM-BIO life science CO., LTD, Shanghai, China). HBV-DNA level was quantitatively measured using a fluorescent PCR detection kit (PG Biotech, Shenzhen, China; Sensitivity: 500 copies/mL) on a LightCycler real-time PCR system (Roche, Basel, Switzerland).

### Ultrasound examination and Definition of response

Abdominal sonographic examination was performed on the Ultrasound instrument of MYLAB90 (ESAOTE, Italy) by senior specialists who were blind to the examinees' medical history and blood test results. The probe frequency was among 3–5 MHz. The diagnosis of fatty liver is as followings: diffusely increased echogenicity (bright) liver where the echogenicity is greater than kidney or spleen; vascular blurring; deep attenuation of ultrasound signal [14].

Primary non-response is defined as <1 log_10_ IU/mL decrease in HBV-DNA level from baseline at 12^wk^ of therapy. Basic virological response is defined as undetectable HBV-DNA in both HBeAg positive and negative CHB patients by real-time PCR assay at time spot of 24^wk^, 48^wk^ and 96^wk^. Virological breakthrough is defined as a confirmed over 1 log_10_ IU/ml increase in HBV-DNA level, compared with the lowest HBV-DNA level during therapy. HBeAg serum conversion is defined as change of HBeAg from positive into negative. ALT normalization is defined as ALT level decreases into within the normal range. Advanced virological response is defined as HBV-DNA clearance, HBeAg serum conversion in HBeAg (+) patients and ALT normalization.

### Statistics

Data were first assessed for normality and log transformed where appropriate. Quantitative variant were expressed as mean ± standard deviation (SD) or median with range once nonnormal distribution was found. Student t test or Mann-Whitney U-test was further applied. For qualitative variant, percentages or frequencies were used and X^2^ test was chosen for further comparison. Binary logistic regression using forward-conditional method was further applied to determine significant variables from univariate analysis. Hepatic steatosis and virological response were appointed as dependent variables and categorized into binary outcomes as absent or present, respectively. SPSS 17.0 was used for statistical analysis through the whole process and p<0.05 was considered statistically significant.

## Results

### General characteristics of subjects

**Figure 1:**
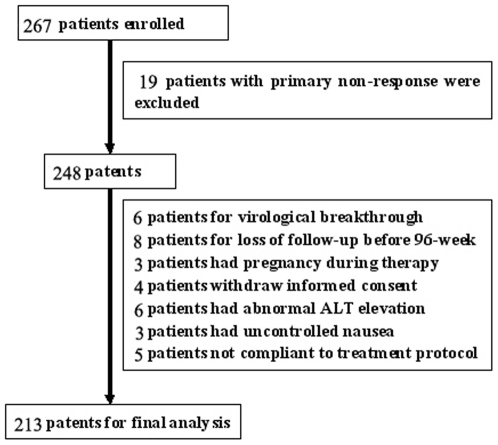
Schematic representation of the selection process for CHB patients receiving initial antiviral therapy.

Totally 267 patents were selected in this study and 54 patients were excluded from final analysis, for the reasons of primary non-response, significant side effect, virological breakthrough, loss of follow-up and so on (Figure 1). Considering the relatively high exclusion rate, we compared baseline demographic, anthropometric and laboratory characteristics between those included and excluded patients. As shown in Table 1, compared with included patients, those excluded patients had significantly lower ALT level (147.38±30.15 vs 183.56±51.02, p = 0.03) but higher ratio of hepatic steatosis (46.3% vs 30.6%, p = 0.04). The other parameters of NAFLD, including BMI, TG, waist circumference and obesity, also showed increased tendency but did not reach statistical significance.

**Table 1:** Baseline demographic, anthropometric, clinical and laboratory characteristics of included and excluded patients.

Parameters	Included patients (n = 213)	Excluded patients (n = 54)	p
Age (y)	37.3±8.09	38.15±8.21	0.77
Sex (Males, n, %)	118 (55.4%)	30 (55.6%)	0.13
BMI (Kg/m^2^)	25.76±4.23	26.37±3.49	0.07
Obesity (n, %)	29 (13.6%)	8 (14.8%)	0.23
Overweight (n, %)	78 (36.6%)	21 (38.9%)	0.14
Waist circumference (cm)	84.21±3.85	85.29±3.07	0.09
Family history of HBV (n, %)	42 (19.7%)	10 (18.5%)	0.18
Hypertension (n, %)	32 (15.0%)	8(14.8%)	0.23
DM (n, %)	13 (6.1%)	3 (5.6%)	0.29
Chol (mmol/L)	4.37±0.35	4.11±0.41	0.23
TG (mmol/L)	1.23±0.37	1.29±0.45	0.09
FBG(mmol/L)	5.06±1.13	5.15±1.41	0.17
ALT (U/L)	183.56±51.02	147.38±30.15	0.03
AST (U/L)	54.63±12.77	51.29±15.17	0.14
ALP (U/L)	72.65±18.71	71.55±18.27	0.25
GGT (U/L)	46.39±14.58	43.89±12.07	0.16
Uric acid (µmol/L)	377.89±50.17	369.04±61.13	0.15
HBV-DNA (10^6^ copies/mL)*	4.51 (0.14–31.5)	4.57 (0.13–32.7)	0.27
HBeAg positive (n, %)	133 (62.4%)	33 (61.1%)	0.13
Hepatic steatosis (n, %)	65 (30.5%)	25 (46.3%)	0.04

* , expressed as median with range, compared by Mann Whitney U test.

All patients entering final analysis were Chinese with average age of 37.3 y, ranging from 19 y to 64 y. The percentage of male was 55.4% and the mean BMI was 25.76 Kg/m^2^. The prevalence of obesity, overweight, DM and hypertension were 13.6%, 36.6%, 6.1% and 15.0%, respectively. The mean waist circumference, ALT, AST, ALP, GGT, TG, Chol, FBG and Uric acid levels were 84.21 cm, 183.56 U/L, 54.63 U/L, 72.65 U/L, 46.39 U/L, 1.23 mmol/L, 4.37 mmol/L, 5.06 mmol/L and 377.89 µmol/L, respectively. The overall percentage of hepatic steatosis was 30.5% (65/271) and the prevalence of HBeAg positive was 62.4%. Patients' HBV-DNA level was varied and nonnormal distributed, with median of 4.51 * 10^6^ copies/mL.

### Association between steatosis and host or viral factors

As shown in Table 2, the distribution of age, sex and family history of HBV infection were not significantly different. Nevertheless, the BMI, waist circumference, serum FBG, TG and uric acid levels as well as the percentages of obesity and overweight were significantly higher in CHB patients with hepatic steatosis (p<0.05). The percentages of DM and hypertension were also higher in CHB patients with hepatic steatosis but did not reach statistical significance (p = 0.26; p = 0.13, respectively). Furthermore, HBV-DNA level and the status of HBeAg positive were equally distributed in CHB patients with and without steatosis. Based on these univariate findings, logistic regression showed that waist circumference, serum TG and uric acid levels were independent factors of hepatic steatosis (Table 3).

**Table 2:** Comparison of baseline demographic, anthropometric, clinical and laboratory characteristics of enrolled patients with and without hepatic steatosis.

Parameters	With Steatosis (n = 65, 30.5%)	Without Steatosis (n = 148, 69.5%)	p
Age (y)	39.56±11.87	39.55±7.83	0.96
Sex (Males, n, %)	32(49.2%)	85(57.4%)	0.08
BMI (Kg/m^2^)	26.35±4.19	24.26±3.41	<0.01
Obesity (n, %)	14 (21.5%)	15 (10.1%)	<0.05
Overweight (n, %)	34 (52.3%)	44 (29.7%)	<0.01
Waist circumference (cm)	86.33±3.31	83.96±3.64	<0.01
Family history of HBV (n, %)	13(20.0%)	35 (23.6%)	0.17
Hypertension (n, %)	12(18.5%)	20 (13.5%)	0.13
DM (n, %)	4 (6.2%)	8(5.4%)	0.26
Chol (mmol/L)	4.46±0.44	4.37±0.32	0.15
TG (mmol/L)	1.53±0.38	1.11±0.39	<0.01
FBG (mmol/L)	5.46±1.37	5.07±0.92	<0.01
ALT (U/L)	171.68±46.23	159.18±45.12	0.12
AST (U/L)	59.66±13.81	56.63±13.13	0.15
ALP (U/L)	71.13±16.32	70.47±18.03	0.82
GGT (U/L)	42.92±14.83	46.05±11.36	1.41
Uric acid (µmol/L)	395.52±44.83	375.26±52.81	<0.01
HBV-DNA (10^6^ copies/mL)*	4.90(0.87–32.0)	4.56(0.15–32.4)	0.18
HBeAg positive (n, %)	38 (58.5%)	95 (64.2%)	0.10

* , expressed as median with range, compared by Mann Whitney U test.

**Table 3:** Multivariate analysis of baseline factors significantly associated with hepatic steatosis and antiviral response.

Factors	Exp (B)	95%CI	SE	p
**Baseline factors associated with hepatic steatosis**				
Waist circumference (cm)	1.160	1.034–1.300	0.058	0.011
TG (mmol/L)	23.814	6.372–88.996	0.673	<0.01
UA (µmol/L)	1.017	1.009–1.025	0.004	<0.01
**Baseline factors associated with antiviral response at 24 week**				
Hepatic steatosis	2.203	1.154–4.204	0.330	0.017
**Baseline factors associated with antiviral response at 48 week**				
Hepatic steatosis	0.333	1.137–4.189	0.184	0.019
**Baseline factors associated with antiviral response at 96 week**				
Hepatic steatosis	2.328	1.162–4.664	0.355	0.017

### Hepatic steatosis as an independent factor for Entecavir treatment failure

The demographic, anthropometric, clinical and laboratory features of Entecavir responders and nonresponders at different time spot were shown in Table S1, S2, S3. The rates of response to Entecavir were 54.9%, 63.8% and 74.2% at 24^wk^, 48^wk^ and 96^wk^, respectively. At 24^wk^, BMI, Waist circumference and prevalence of hepatic steatosis were significantly higher in nonresponders than in responders (p<0.05, Table 4). Using multivariate regression, hepatic steatosis was confirmed as an independent factor for basic virological response (p = 0.017, Table 3). Other metabolic features including obesity and overweight did not show significant difference between those patients. Viral factors including HBV-DNA level and percentage of HBeAg positive decreased in nonresponders, but the extent did not reach statistical significance.

**Table 4:** List of independent factors significantly associated with nonresponse to Entecavir at 24, 48 and 96 weeks (revealed by Univariate analysis).

Variables	responders	nonresponders	p
**24 week**			
BMI (Kg/m^2^)	24.33±3.70	25.65±3.78	0.02
Waist circumference (cm)	84.10±3.12	85.43±3.45	0.02
Hepatic steatosis (n, %)	27(23.1%)	38(39.6%)	0.02
**48 week**			
BMI (Kg/m^2^)	24.45±3.64	25.75±3.91	0.03
Waist circumference (cm)	84.28±3.80	85.42±3.46	0.04
Hepatic steatosis	34(25.0%)	32(41.6%)	0.02
**96 week**			
Waist circumference (cm)	84.34±3.82	85.68±3.22	0.03
HBV-DNA (10^6^ copies/mL)*	4.87(0.15–32.0)	4.05(0.21–32.40)	0.04
Hepatic steatosis	41(26.0%)	24(43.6%)	0.01

* , expressed as median with range, compared by Mann Whitney U test.

At 48^wk^, the factors significantly increased in nonresponders were also BMI, waist circumference and hepatic steatosis (Table 4), further revealed the influence of central obesity in virological response. However, only hepatic steatosis was confirmed as an independent factor under multivariate logistic regression (p = 0.019, Table 3). As similar as at 24^wk^, there were no significant differences in ALT, AST, ALP, GGT, Chol, TG, FBG, uric acid, DM, hypertension, family history of HBV, status of HBeAg positive and HBV-DNA level between responders and nonresponders. At 96^wk^, waist circumference and percentage of hepatic steatosis continued to be significantly higher in nonresponders (Table 4). Nevertheless, the increased level of BMI and percentage of obesity did not reach statistical significance. Intriguingly, HBV-DNA level was significantly lower in those nonresponders (p = 0.04). Nevertheless, of those factors, only hepatic steatosis was proved to be an independent factor under multivariate logistic regression (p = 0.017, Table 3).

### Association between hepatic steatosis and advanced virological response

We further investigated the association between hepatic steatosis and advanced virological response by post hoc analysis of our prospectively enrolled cohorts. Different from previous nested case control study, we separately compared the rates of HBV-DNA clearance, HBeAg seroconversion and ALT normalization between CHB patients with and without hepatic steatosis at separate time spot. As shown in Table 5, the rate of HBV-DNA clearance was significantly increased as 58.8%, 67.6% and 77.7% at 24^wk^, 48^wk^ and 96^wk^ in patients without hepatic steatosis. The rate of ALT normalization was higher in patents without steatosis throughout the whole time spot, but reached statistical significance from 48^wk^. In contrast, there were no significant differences in HBeAg seroconversion between two groups at 24^wk^, 48^wk^ and 96^wk^.

**Table 5:** Advanced virological response to Entecavir therapy in CHB patients with and without hepatic steatosis.

Variables	With steatosis (n = 65, 30.5%)	Without steatosis (n = 148, 69.5%)	p
**24 week**			
HBV-DNA clearance (n, %)	31(47.7%)	87 (58.8%)	0.01
HBeAg seroconversion (n, %)	10 (15.4%)	23(15.5%)	0.28
ALT normalization (n, %)	26 (40.0%)	65 (43.9%)	0.11
**48 week**			
HBV-DNA clearance (n, %)	35(53.8%)	100 (67.6%)	0.04
HBeAg seroconversion (n, %)	12 (18.5%)	33(22.3%)	0.11
ALT normalization (n, %)	38 (58.5%)	105 (70.9%)	0.04
**96 week**			
HBV-DNA clearance (n, %)	42 (64.6%)	115 (77.7%)	0.04
HBeAg seroconversion (n, %)	16 (24.6%)	42 (28.4%)	0.13
ALT normalization (n, %)	49 (75.4%)	129 (87.2%)	0.03

## Discussion

Nowadays, accumulated evidences showed that hepatic steatosis is a common phenomenon in CHB patients, as we verified in current study. We found that the prevalence of hepatic steatosis was 30.5% in CHB patients, in agreement with most published reports and higher than that in general population of 10%–24% [15]. However, as patients included in this study were not randomly chosen and those CHB patients with ALT level <2 ULN were excluded, our findings may not represent the prevalence of hepatic steatosis in general CHB patients. Besides, the primary nonresponse in this study was relatively higher (7.1%, Figure 1) than previous report [16], which may be due to low patients' compliance to drug administration. In addition, hepatic steatosis and inflammation could also result in ALT increment, which may mask real ALT change caused by HBV activation and thus misclassified CHB patients into antiviral therapy. Therefore, should we increase the criteria of antiviral therapy in CHB patients with hepatic steatosis? Should we treat NAFLD before selecting CHB patients with NAFLD for anti-HBV therapy? Those interesting questions were raised from this study but needed further investigation.

The demographic data were equally distributed between CHB patients with and without hepatic steatosis (Table 2), showing high inter-group balance. In this study, we found a significantly higher BMI, waist circumference, uric acid and TG levels as well as percentages of obesity and overweight in CHB patients with hepatic steatosis. However, HBV-DNA level and status of HBeAg positive did not show significant difference between those groups. These findings supported the hypothesis that hepatic steatosis in CHB patients is associated with metabolic factors than viral factors. Since recognized as the hepatic manifestation of metabolic syndrome, CHB patients with hepatic steatosis were supposed to have higher percentages of DM and hypertension but the difference in our study was not statistically significant (Table 2). This might be due to the low amount of patients with these two ailments. However, the FBG level in hepatic steatosis group was significantly higher, supporting the coexistence of dys-regulated glucose metabolism. Previous works found that BMI and TG were independent factors for hepatic steatosis [8], [17] while our findings showed that waist circumference was also associated with hepatic steatosis (Table 3). This result complemented previous findings that BMI and waist circumference were associated with NASH [18]. In addition, we also found uric acid as an independent risk factor for hepatic steatosis, confirming our latest findings that uric acid level was significantly associated with NAFLD [19].

Currently, Entecavir has been confidently considered as first-line monotherapy of CHB, for its potent HBV inhibition ability and a high barrier to resistance [20]. Nevertheless, the effect of anti viral drugs in CHB patients with hepatic steatosis was rarely reported. There was only one article showing none impact of hepatic steatosis on the outcome of peg-α-interferon treatment in CHB patients [21]. Therefore, this study firstly reported the negative effect of hepatic steatosis on Entecavir treatment failure in CHB patients. Such effect is biologically possible, as cellular fat accumulation may decrease the contact area between drugs and hepatocytes, causing reduced bioavailability of Entecavir [22]. Besides, diminished activity of hepatic cytochromes in steatotic hepatocytes may also hamper drug metabolism [23]. In patients with hepatitis C, insulin resistance and obesity coexisted with hepatic steatosis may lead to dysfunction of cellular immune function [24], which might be also true in CHB patients with hepatic steatosis.

To further analyze the association between hepatic steatosis and advanced virological response, we retrospectively separated CHB patients into groups with and without hepatic steatosis and further investigated the overall difference of antiviral effect from 24^wk^ to 96^wk^. This in-depth analysis showed a significant effect of hepatic steatosis on HBV-DNA clearance and ALT normalization (Table 5). Our finding was of clinical importance as it may change current mode of antiviral therapy in CHB patients with hepatic steatosis. Moreover, it is rational to further investigate the effect of treating hepatic steatosis on HBV antiviral therapy. We have carried out an RCT (ClinicalTrials: NCT01148576) by using Entecavir with essentiale or vitamin E to treat CHB patients with hepatic steatosis. Besides, we did a preliminary multivariant analysis on CHB patients without steatosis and found ALT was significantly associated with Entecavir treatment failure at 24^wk^ (p = 0.03), in contrast with our previous finding (Table 3). This result supports our hypothesis that hepatic steatosis caused ALT elevation may mask real HBV activation caused ALT elevation. Nevertheless, such association did not show statistic significance at 48^wk^ and 96^wk^, which needs further study with larger subjects.

Admittedly, there are many shortages of this study. Firstly, serum insulin level was not detected and insulin resistance was previously shown to impair response to peginterferon plus ribavirin in CHC patients [25]. Secondly, we did not analyze HBV genotypes, where it is plausible that some genotypes exhibit “steatoviruses” characteristics, as shown in HCV genotype 3 [26]. Thirdly, we didn't use liver biopsy in determining hepatic steatosis, as this test is invasive and may cause both minor and major complications [27]. In this study, as elevated HBV-DNA and ALT levels were found in all CHB patients, there might be of less value to preclude other causes of liver damage by biopsy. In contrast, the non-invasive hepatic ultrasound showed a sensitivity over 80% and specificity over 90% for steaosis [28]. Therefore, hepatic ultrasound was used to detect steatosis in daily clinical practice for the strength of the least expensive and most convenient modality [29], as was done in our study. Fourthly, we only analyzed Chinese patients and our results need verification in other ethnics. Finally, we have not observed any resistance to Entecavir until the end of this study, which may be due to the relatively short observation period and low amount of subjects. Nevertheless, those weaknesses could not overwhelm the original findings of this prospective unmatched nested case control study, which may change the standard therapy of CHB patients with NAFLD.

In summary, this study demonstrated, for the first time, that hepatic steatosis is significantly associated with Entecavir treatment failure in CHB patients. Current study also confirmed the association of metabolic factors with hepatic steatosis. These novel results raised the issue on developing specific treatment strategy in CHB patients with NAFLD, which needs investigation in the future. Further studies should also focus on the molecular mechanism of steatosis on nonresponse to Entecavir and other antiviral drugs.

## Supporting Information

Table S1 **Univariate analysis of factors associated with nonresponse to Entecavir at 24 week.**
(DOC)

Table S2 **Univariate analysis of factors associated with nonresponse to Entecavir at 48 week.**
(DOC)

Table S3 **Univariate analysis of factors associated with nonresponse to Entecavir at 96 week.**
(DOC)
