# Supplementary material for: Association between Hepatic Steatosis and Entecavir Treatment Failure in Chinese Patients with Chronic Hepatitis B
Source: PLoS One. 2012 Mar 30;7(3):e34198. doi: 10.1371/journal.pone.0034198 (PMC3316632; doi:10.1371/journal.pone.0034198)
Supplement: Table S1 — Univariate analysis of factors associated with nonresponse to Entecavir at 24 week. (DOC) [file pone.0034198.s001.doc]

**Table S1, Univariate analysis of factors associated with nonresponse to Entecavir at 24 week**

Variables responders (117, 54.9%) nonresponders (96, 45.1%) p

Age (y) 39.49±9.46 39.80±9.03 0.83

Sex (Males, n, %) 54(54.5%) 43(53.8%) 0.92

BMI (Kg/m^2^) 24.33±3.70 25.65±3.78 0.02

Obesity (n, %) 15(15.2%) 10(12.5%) 0.61

Overweight (n, %) 33(33.3%) 33(41.3%) 0.28

Waist circumference (cm) 84.10±3.12 85.43±3.45 0.02

Family history of HBV 19(19.2%) 16(20.0%) 0.89

Hypertension (n, %) 15(15.2%) 11(13.8%) 0.79

DM (n, %) 5(5.1%) 6(7.5%) 0.50

Chol (mmol/L) 4.39±0.40 4.43±0.37 0.47

TG (mmol/L) 1.26±0.40 1.33±0.38 0.20

FBG (mmol/L) 5.12±1.01 5.21±1.24 0.62

ALT (U/L) 161.45±43.43 164.28±48.62 0.68

AST (U/L) 58.03±13.73 56.99±12.90 0.60

ALP (U/L) 69.97±16.82 71.54±18.33 0.55

GGT (U/L) 44.79±12.64 45.46±12.58 0.72

Uric acid (μmol/l) 376.36±58.59 374.70±64.70 0.86

HBV-DNA (10^6^ copies/mL)* 4.87 (0.15-32.0) 4.31 (0.15-32.4) 0.14

HBeAg positive (n, %) 67(67.7%) 44(55.0%) 0.08

Hepatic steatosis (n, %) 27(23.1%) 38(39.6%) 0.02

*, expressed as median with range, compared by Mann Whitney U test.
