# Supplementary material for: Association between Hepatic Steatosis and Entecavir Treatment Failure in Chinese Patients with Chronic Hepatitis B
Source: PLoS One. 2012 Mar 30;7(3):e34198. doi: 10.1371/journal.pone.0034198 (PMC3316632; doi:10.1371/journal.pone.0034198)
Supplement: Table S2 — Univariate analysis of factors associated with nonresponse to Entecavir at 48 week. (DOC) [file pone.0034198.s002.doc]

**Table S2, Univariate analysis of factors associated with nonresponse to Entecavir at 48 week**

Variables responders (136, 63.8%) nonresponders (77, 36.2%) p

Age (y) 39.82±9.26 39.31±9.28 0.73

Sex (Males, n, %) 66(57.9%) 31(47.7%) 0.19

BMI (Kg/m^2^) 24.45±3.64 25.75±3.91 0.03

Obesity (n, %) 16(14.0%) 9(13.8%) 0.97

Overweight (n, %) 40(35.1%) 26(40.0%) 0.51

Waist circumference (cm) 84.28±3.80 85.42±3.46 0.04

Family history of HBV 21(18.4%) 14(21.5%) 0.61

Hypertension (n, %) 16(14.0%) 10(15.4%) 0.81

DM (n, %) 6(5.3%) 5(7.7%) 0.52

Chol(mmol/L) 4.39±0.39 4.45±0.38 0.33

TG (mmol/L) 1.26±0.40 1.34±0.36 0.21

FBG(mmol/L) 5.14±1.00 5.20±1.29 0.76

ALT (IU/L) 161.12±43.18 165.51±50.06 0.54

AST (IU/L) 57.10±13.34 58.38±13.40 0.54

ALP (IU/L) 71.31±17.87 69.55±16.85 0.52

GGT (IU/L) 44.96±12.97 45.32±11.96 0.85

Uric acid (μmol/L) 371.82±59.91 382.28±63.38 0.27

HBV-DNA (10^6^ copies/mL) * 4.92(0.15-32. 0) 4.19(0.21-32.4) 0.09

HBeAg positive (n, %) 75(65.8%) 36(55.4%) 0.17

Hepatic steatosis 34(25.0%) 32(41.6%) 0.02

*, expressed as median with range, compared by Mann Whitney U test.
