# Supplementary material for: Association between Hepatic Steatosis and Entecavir Treatment Failure in Chinese Patients with Chronic Hepatitis B
Source: PLoS One. 2012 Mar 30;7(3):e34198. doi: 10.1371/journal.pone.0034198 (PMC3316632; doi:10.1371/journal.pone.0034198)
Supplement: Table S3 — Univariate analysis of factors associated with nonresponse to Entecavir at 96 week. (DOC) [file pone.0034198.s003.doc]

**Table S3, Univariate analysis of factors associated with nonresponse to Entecavir at 96 week**

Variables responders (158, 74.2%) nonresponders (55, 25.8%) p

Age (y) 39.72±9.49 39.37±8.59 0.82

Sex (Males, n, %) 77(57.9%) 20(43.5%) 0.09

BMI (Kg/m^2^) 24.73±3.81 25.45±3.69 0.27

Obesity (n, %) 20(15.0%) 5(15.2%) 0.44

Overweight (n, %) 49(36.8%) 17(37.0%) 0.99

Waist circumference (cm) 84.34±3.82 85.68±3.22 0.03

Family history of HBV 24(18.0%) 11(23.9%) 0.39

Hypertension (n, %) 21(15.8%) 5(10.9%) 0.41

DM (n, %) 8(6.0%) 3(6.5%) 0.90

Chol (mmol/L) 4.41±0.39 4.43±0.38 0.77

TG (mmol/L) 1.27±0.39 1.35±0.38 0.18

FBG (mmol/L) 5.20±1.14 5.03±1.05 0.38

ALT (IU/L) 161.66±41.98 165.76±55.52 0.60

AST (IU/L) 57.73±13.71 57.09±12.33 0.78 ALP (IU/L) 70.71±17.42 70.57±17.86 0.96 GGT (IU/L) 44.26±12.66 47.50±12.18 0.13 Uric acid (μmol/L) 373.86±61.09 380.70±62.01 0.52 HBV-DNA (10^6^ copies/mL) * 4.87(0.15-32.0) 4.05(0.21-32.40) 0.04 HBeAg positive (n, %) 84(63.2%) 27(58.7%) 0.59 Hepatic steatosis 41(26.0%) 24(43.6%) 0.01

*, expressed as median with range, compared by Mann Whitney U test.
